# Occupational quartz and particle exposure affect systemic levels of inflammatory markers related to inflammasome activation and cardiovascular disease

**DOI:** 10.1186/s12940-023-00980-1

**Published:** 2023-03-13

**Authors:** Alexander Hedbrant, Christopher Engström, Lena Andersson, Daniel Eklund, Håkan Westberg, Alexander Persson, Eva Särndahl

**Affiliations:** 1 School of Medical Sciences, Faculty of Medicine and Health, Örebro University, 701 82 Örebro, Sweden; 2 Inflammatory Response and Infection Susceptibility Centre (iRiSC), Faculty of Medicine and Health, Örebro University, 701 82 Örebro, Sweden; 3 Division of Mathematics and Physics, The School of Education, Culture and Communication, Mälardalen University, Box 883, 721 23 Västerås, Sweden; 4 Department of Occupational and Environmental Medicine, Örebro University Hospital, 701 85 Örebro, Sweden

**Keywords:** Inflammation, Occupational exposure, Silica, PM, NLRP3 inflammasome, CVD biomarkers

## Abstract

**Background:**

The inflammatory responses are central components of diseases associated with particulate matter (PM) exposure, including systemic diseases such as cardiovascular diseases (CVDs). The aim of this study was to determine if exposure to PM, including respirable dust or quartz in the iron foundry environment mediates systemic inflammatory responses, focusing on the NLRP3 inflammasome and novel or established inflammatory markers of CVDs.

**Methods:**

The exposure to PM, including respirable dust, metals and quartz were determined in 40 foundry workers at two separate occasions per worker. In addition, blood samples were collected both pre-shift and post-shift and quantified for inflammatory markers. The respirable dust and quartz exposures were correlated to levels of inflammatory markers in blood using Pearson, Kendall τ and mixed model statistics. Analyzed inflammatory markers included: 1) general markers of inflammation, including interleukins, chemokines, acute phase proteins, and white blood cell counts, 2) novel or established inflammatory markers of CVD, such as growth/differentiation factor-15 (GDF-15), CD40 ligand, soluble suppressor of tumorigenesis 2 (sST2), intercellular/vascular adhesion molecule-1 (ICAM-1, VCAM-1), and myeloperoxidase (MPO), and 3) NLRP3 inflammasome-related markers, including interleukin (IL)-1β, IL-18, IL-1 receptor antagonist (IL-1Ra), and caspase-1 activity.

**Results:**

The average respirator adjusted exposure level to respirable dust and quartz for the 40 foundry workers included in the study was 0.65 and 0.020 mg/m^3^, respectively. Respirable quartz exposure correlated with several NLRP3 inflammasome-related markers, including plasma levels of IL-1β and IL-18, and several caspase-1 activity measures in monocytes, demonstrating a reverse relationship. Respirable dust exposure mainly correlated with non-inflammasome related markers like CXCL8 and sST2.

**Conclusions:**

The finding that NLRP3 inflammasome-related markers correlated with PM and quartz exposure suggest that this potent inflammatory cellular mechanism indeed is affected even at current exposure levels in Swedish iron foundries. The results highlight concerns regarding the safety of current exposure limits to respirable dust and quartz, and encourage continuous efforts to reduce exposure in dust and quartz exposed industries.

**Supplementary Information:**

The online version contains supplementary material available at 10.1186/s12940-023-00980-1.

## Introduction

The aim of this study was to determine effects on the inflammatory response mediated by particulate matter (PM) exposure in the iron foundry environment. The potential of PM to induce inflammatory reactions is well established, and the inflammatory potential of different PM is one key factor to determine their toxicological effects in humans. Exposure to airborne PM is a major concern for human health, and exposures to ambient and household PM with an aerodynamic diameter of 2.5 µm or less (PM_2.5_) is estimated to cause 7 million deaths annually [1]. The mechanisms regarding PM exposure and their harmful effects in the lungs include direct toxicity of the particles per se, as well as damage due to the inflammatory responses elicited in response to PM exposure. The effects of PMs are not restricted to the lung tissues, as particles in the nano-sized range as well as the inflammatory mediators generated at the site of exposure may enter the systemic circulation and could thereby exert both toxic and inflammatory effects by direct interaction with distal tissues and organs [2].

Chronic, sub-clinical low-grade inflammation is considered to play a central role in the etiology of several non-communicable diseases, including cardiovascular diseases (CVD), obesity- [3] and age-related diseases [4]. PM exposure contributes to increased risk of CVDs and diseases of the respiratory system [5, 6], and increasing evidence indicate that the systemic low-grade inflammation induced by PMs exposure is a main mechanism of these diseases [2, 7–9]. Importantly, not only chronic exposure to PM, but also acute short-term increases in PM exposure, has been associated with health risks; mainly linked to cardiovascular events, including hospitalization and deaths [10–12]. Short-term increases in ambient PM exposure have furthermore been found to induce acute biological effects, including increases in inflammatory mediators, changes in small metabolites, and effects on heart rate and blood pressure; processes central for the intricate etiology of CVD [9, 10, 13, 14].

The associations between PM exposure and effects on systemic inflammation and inflammatory diseases, including CVDs, are best characterized for ambient PM. However, similar evidence is also emerging for occupational PM exposures. For example, a recent study demonstrated increased risk of rheumatoid arthritis due to various occupational inhalable agents, including e.g., quartz dust and welding fumes [15].

The iron foundry environment is a milieu with high levels of PM, generally far above the levels found in outdoor environments; however, the chemical composition of the PM and their size distribution found in foundries differ to ambient PM and could therefore differ also in toxicological aspects. Iron foundry dust contains high levels of quartz (crystalline silica), which is a well-known hazardous particle that can cause inflammation in the respiratory tract and lung diseases, such as silicosis. Additionally, long-term quartz exposure has also been linked to CVD for workers in foundries [16, 17], metal mines, and potteries [18, 19].

Quartz particles are potent at inducing inflammatory mediators in vitro*,* and are particles known to activate the NLRP3 inflammasome complex that upon assembly activates the protease caspase-1, which in turn cleaves the pro-forms of interleukin (IL)-1β and IL-18 to their active forms. However, whether PM and quartz exposures at current exposure levels in the iron foundry environment cause inflammatory effect in exposed workers has not been described.

In this study, PM presented in the foundries was characterized by metal and quartz content, and the respirable PM and quartz exposures present at two Swedish iron foundries were analyzed with the aim to correlate exposures to the biological host response. Accordingly, the impact of quartz and PM exposure in iron foundry workers on inflammatory markers were broadly classified into three categories: 1) general markers of inflammation, 2) novel inflammatory biomarkers of CVD, and 3) markers of NLRP3 inflammasome activation.

## Results

### Exposures to PM and quartz in the iron foundries

**Fig. 1:**
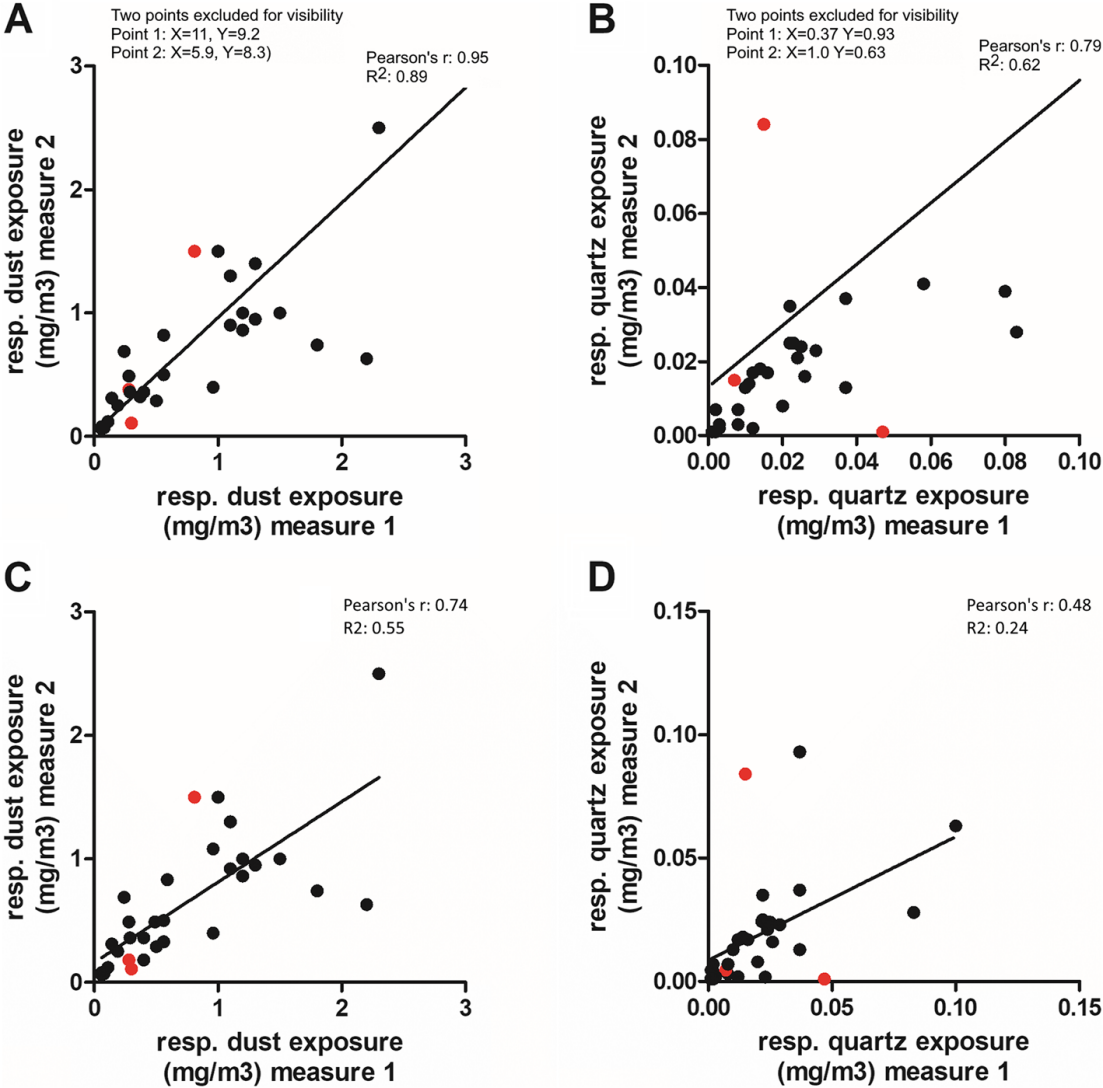
Within/between worker variation for the 32 individuals that were sampled during two separate measurements. Non-respirator adjusted exposure to respirable dust (**A**), respirable quartz (**B**), respirator adjusted exposure for respirable dust (**C**), and respirable quartz (**D**). Three workers, indicated by red dots, changed work tasks between the two measurements; two changed work tasks in the production line and one changed from production line to product controller. Two extreme high values were not visually shown in the figure, as noted in the figure, for readability of the lower exposures

The exposures to airborne PM, respirable quartz, and inhalable metal particles are shown in Table 1. For all measurement days, that were part of this study, the foundries had a normal production without major breaks. The personal average respirable dust and quartz exposures, when adjusted for respirator use, were 0.65 and 0.020 mg/m^3^, respectively. The highest respirator adjusted values for respirable dust and quartz were 2.5 and 0.1 mg/m^3^, respectively, which are levels equivalent to the current Swedish occupational exposure limits (OELs). The PM exposures separated for different workstations is not presented in the table; however, the PM levels were highest in the blasting/fettling areas (mean respirable dust 4.9 mg/m^3^, *n* = 8). In these areas, the workers used protective equipment for the vast majority of the time, thus the respirator adjusted exposures in the blasting/fettling areas were similar to those levels found for individuals performing work in the casting/mold making areas (*i.e.* adjusted average exposures around 0.8 mg/m^3^ for both these groups). As a result, the highest average respirable dust exposure, when adjusted for respirator use, was found at the melting departments (1.0 mg/m^3^). Regarding respirable quartz, the highest average exposure was observed in the blasting/fettling areas also when adjusting for respirator use (0.045 mg/m^3^). The within/between worker variation in exposure levels comparing the two measurements, are shown in Fig. 1. The intra-individual exposures had a strong correlation for the two separate measures regarding respirable dust, with a Pearson’s r value of 0.95 (*p* < 0.0001) or 0.74 (*p* < 0.0001) for non-adjusted and respirator adjusted exposures, respectively. For respirable quartz, the Pearson’s r correlation was weaker (0.79, *p* < 0.0001) or 0.48 (p 0.005) for non-adjusted and respirator adjusted exposures, respectively.

**Table 1:** Particle exposure measures

**Personal measurements (mg/m**^**3**^**)**	N	AM	SD	GM	GSD	Min	Max
Respirable dust	72	1.3	2.05	0.57	3.5	0.058	11
Respirable dust, resp. adj	72	0.65	0.57	0.43	2.8	0.058	2.5
Respirable quartz	72	0.066	0.18	0.016	4.6	0.0012	1.0
Respirable quartz, resp. adj	72	0.020	0.022	0.011	3.4	0.0011	0.10
**Stationary measurements (mg/m**^**3**^**)**							
Inhalable dust	24	4.7	6.62	3.1	2.8	0.77	35
Respirable dust	24	1.14	0.95	0.83	2.2	0.22	3.7
Respirable quartz	24	0.026	0.032	0.014	3	0.0018	0.14
PM_10_	24	2.74	2.47	2.1	2.1	0.54	11.7
PM_2.5_	24	1.52	2.39	0.81	2.8	0.16	11.7
PM1 (ultrafine)	24	0.40	0.41	0.26	2.6	0.040	1.84
Total particle area							
(A-trak) µm^2^/cm^3^	24	560	576	311	3.3	32	2600
Particle number							
(P-trak)/cm^3^	24	134 774	111 109	90 245	2.6	15 800	467 600

Different PM exposure measures (8-h time weight average levels) measured at two Swedish iron foundries. The personal measurements include 72 measurements from 40 individuals (32 individuals sampled twice). The stationary measurements were positioned at the casting/mold making site (*n* = 10), core making (*n* = 4), sand preparation (*n* = 3), melting (*n* = 3), shake out (*n* = 1), fettling (*n* = 2), and feedstock area (*n* = 1)

*GM* Geometric mean, *GSD* Geometric standard deviation, *AM* Arithmetic mean, *SD* Arithmetic standard deviation, *resp. adj*. Respirator adjusted exposure values

### Dust characterization

To get better understanding on the characteristics of the PM exposures at the iron foundries, the inhalable dust, collected from the stationary measurements, were analyzed for metal content, whereas a particle impactor was used to analyze the size distribution of PM_2.5_ and to determine the quartz content for the different fractions. As shown in Table 2, the metal content of the inhalable dust varied considerably, constituting 5–45% of the total mass. Iron was the main metal found in the inhalable dust, followed by magnesium and aluminum when comparing the geometric means. All of the metals analyzed had mean or median exposure levels well below the Swedish OELs. One measurement in the fettling/blasting area was most likely above the Swedish OEL for iron, copper, and chromium, when considering that the OEL is set for other dust fractions than the analyzed inhalable fraction. However, the workers in our study did always use protective equipment while performing these work tasks.

**Table 2:** Metal analysis of the inhalable dust fraction

*N* = 22 dust filters	AM	SD	GM	GSD	Min	Max	Swedish OEL	OEL dust fraction
Inhalable dust (µg/m^3^)	4 529	6 879	2 947	2.2	805	34 100	5 000	Inhalable
Metals, % of total mass	21	12	18	1.9	5	45		
**Metals (µg/m**^**3**^**)**								
Iron (Fe)	976	2 831	264	4	34	13 500	3 500	Respirable
Magnesium (Mg)	157	164	70	4.6	5.1	480	N/A	
Zink (Zn)	69	180	7.9	9.9	0.41	840	N/A	
Aluminum (Al)	64	75	44	2.2	13	345	5 000	Total dust
Calcium (Ca)	36	61	19	2.8	6.0	295	N/A	
Chromium (Cr)	32	135	0.63	13	< 0.061	635	500	Total dust
Manganese (Mn)	14	27	3.9	4.9	0.50	101	200	Inhalable
Barium (Ba)	7.9	18	2.8	3.8	0.41	89	500	Total dust
Copper (Cu)	5.2	13	1.1	6	0.064	62	10	Respirable
Lead (Pb)	3.7	6.5	0.76	7.1	0.046	22	100	Inhalable
Nickel (Ni)	2.1	4.1	0.47	6.3	< 0.12	19	500	Total dust
Antimony (Sb)	0.48	1.1	0.16	3.8	< 0.095	5.3	250	Inhalable
Vanadium (V)	0.42	1.3	0.09	4.2	< 0.059	6.2	200	Total dust
Molybdenum (Mo)	0.33	0.76	0.094	4.2	< 0.059	3.5	10 000	Total dust
Cobalt (Co)	0.21	0.54	0.073	3.2	< 0.059	2.6	20	Inhalable
Arsenic (As)	0.11	0.14	< 0.14	1.9	< 0.12	0.70	10	Inhalable
Cadmium (Cd)	0.073	0.10	< 0.058	2.3	< 0.056	0.36	4	Inhalable
Tallium (Tl)	0.0051	0.0052	< 0.0066	1.8	< 0.0056	0.024	N/A	

Considering the OEL dust fractions, the total dust fraction is comparable to the inhalable fraction (approx. 100 µm in aerodynamic diameter and smaller), whereas the respirable fraction represents particles approximately 4 µm and smaller. The dust filters were placed accordingly: casting/mold making site (*n* = 10), core making (*n* = 4), sand preparation (*n* = 3), melting (*n* = 2), fettling (*n* = 2), and feedstock area (*n* = 1). N/A indicate that no Swedish OELs is set for the given element

*AM* Arithmetic mean, *SD* Arithmetic standard deviation, *GM* Geometric mean, *GSD* Geometric standard deviation, *OEL* Occupational exposure limit, *N/A* Not applicable

**Fig. 2:**
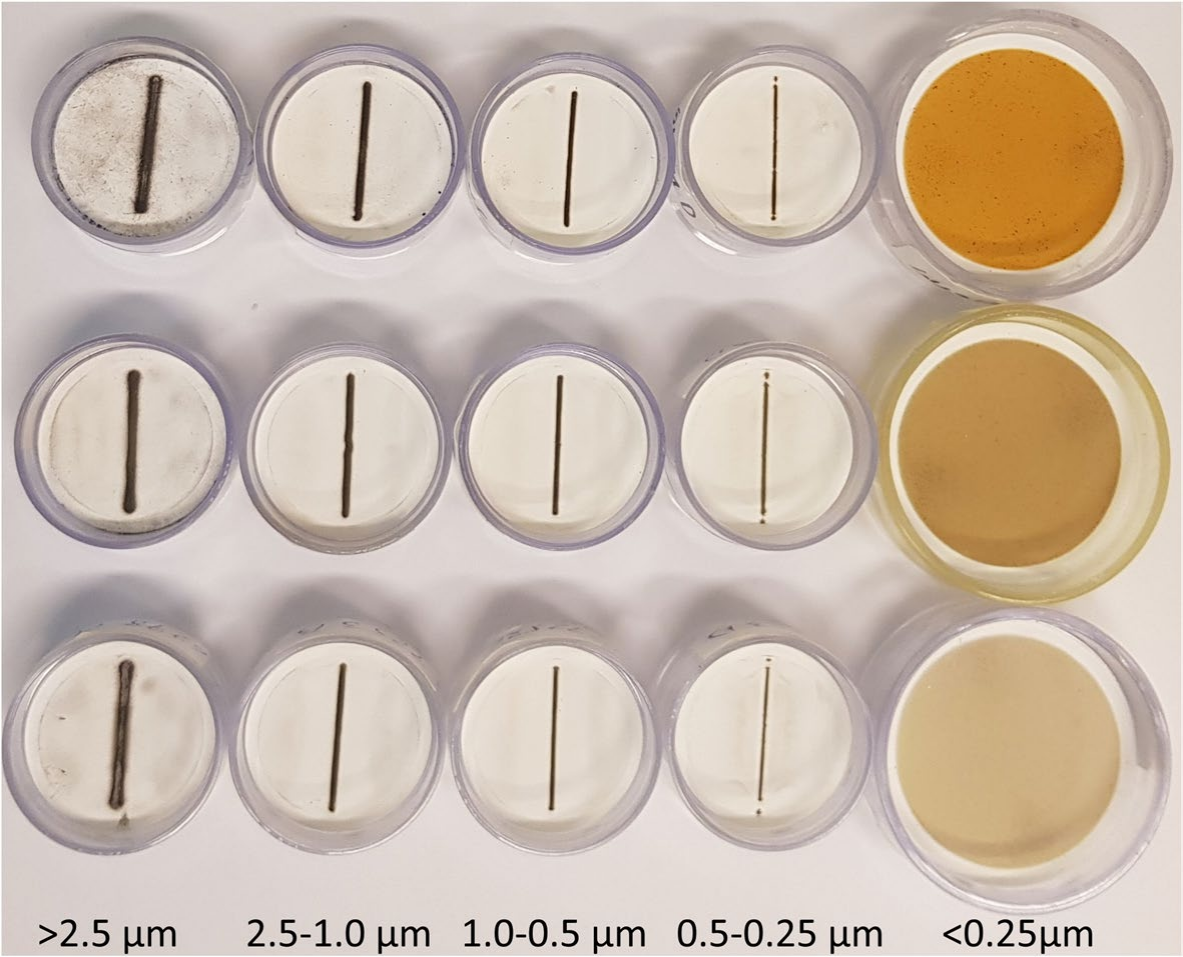
Impactor filters with particles from different size fractions (< 0.25—> 2.5 µm; from three separate measurements, where each row represent filters from a separate measurement occasion) collected in two Swedish iron foundries. A distinct brown/red color is observed in the smallest fraction (< 0.25 µm)

The particle size distribution and quartz content of the collected dust was measured with a particle impactor (Table 3). The results demonstrate that 20% of the total mass on average is in the < 0.25 µm range, *i.e.*, nano-sized particles. However, the quartz content is highest at the largest particle fraction (> 2.5 µm), and steadily decreases with smaller particle fractions, constituting 1% of the mass at the smallest fraction (< 0.25 µm). The color of the particles is distinctly different in the < 0.25 µm fraction compared to the other size fractions (Fig. 2), indicating different particle composition in this fraction, with a higher content of *e.g.*, iron oxides.

**Table 3:** Size distribution of the respirable fraction, and quartz content of the different size ranges

	% Of total mass (*n* = 8)					Quartz content (%) (*n* = 8)				
PM size (µm)	Median	Mean	SD	Min	Max	Median	Mean	SD	Min	Max
> 2.5	43.4	44.9	9.6	34.7	58.6	4.7	4.6	2.0	1.7	7.8
2.5–1.0	18.0	18.5	5.5	11.4	27.6	3.7	3.8	1.4	2.1	5.8
1.0–0.5	9.0	9.4	3.2	4.2	13.5	1.8	2.3	1.8	0.5	5.7
0.5–0.25	6.0	6.5	3.1	2.0	12.8	1.0	1.5	1.4	0.2	3.9
< 0.25	19.5	20.7	7.0	8.6	32.1	1.2	1.1	0.7	0.1	2.4

*PM* Particulate matter, *SD* Standard deviation

### Pre-shift and post-shift levels of inflammatory markers

**Fig. 3:**
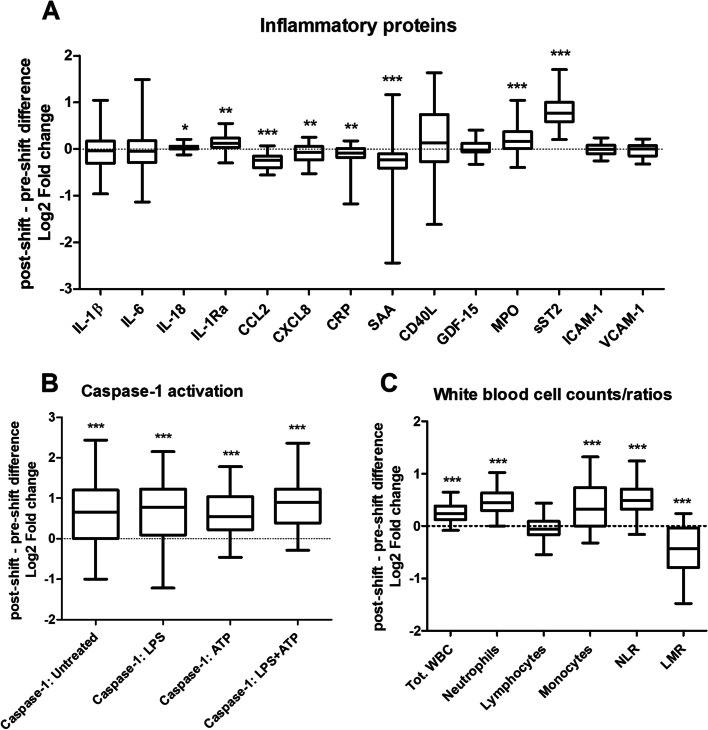
Over-shift difference in inflammatory markers. The data represent average log2 fold change difference in inflammatory markers in blood, when comparing post-shift with pre-shift levels for the 40 foundry workers included in the study. **A** Inflammatory mediators measured in plasma, **B** Percent caspase-1 (inflammasome) activated monocytes following indicated ex vivo treatment, **C** WBC counts and cell ratios. Whiskers of the Box plots indicate min and max values. The asterisks indicate significant over-shift difference in inflammatory marker levels, calculated using the Wilcoxon signed-rank test. * *P*-value < 0.05, ** < 0.01, *** < 0.001. LPS: lipopolysaccharide, ATP: adenosine triphosphate, NLR: neutrophil–lymphocyte ratio, LMR: lymphocyte-monocyte ratio

The studied inflammatory markers included plasma concentration of inflammatory proteins, WBC counts, and ex vivo inflammasome activation in monocytes from study participants´ whole blood. Differences in the pre- and post-shift levels were observed for most of the measured inflammatory markers (Fig. 3 A-C), indicating a diurnal variation and/or work-related effect, such as particle exposure. Significant differences were observed for 8/14 detected plasma proteins (Fig. 3 A), for all reported WBC counts except for lymphocytes (Fig. 3 B), and for all measures of caspase-1 activity (Fig. 3 C). The total WBC count as well as the neutrophil and monocyte counts were found to be increased in the afternoon, while the number of eosinophils were slightly reduced at this time point. A significantly lower plasma concentration was found in the post-shift samples for four of the measured proteins (CRP, SAA, CXCL8, and CCL2), whereas four proteins were significantly higher in concentration in the post-shift samples when compared to pre-shift levels (IL-1Ra, IL-18, sST2, and MPO). Regarding ex vivo inflammasome activation measured as caspase-1 enzymatic activity in monocytes, a higher percentage of caspase-1 activated cells was observed in the post-shift samples for all experimental conditions, including non-treated cells as well as in cells treated with the inflammasome stimuli LPS and/or ATP. The pre- and post-shift inflammatory marker levels, the over-shift differences, and their *p*-values are shown in Additional file 1: Table S1.

### Correlation network of studied inflammatory markers

**Fig. 4:**
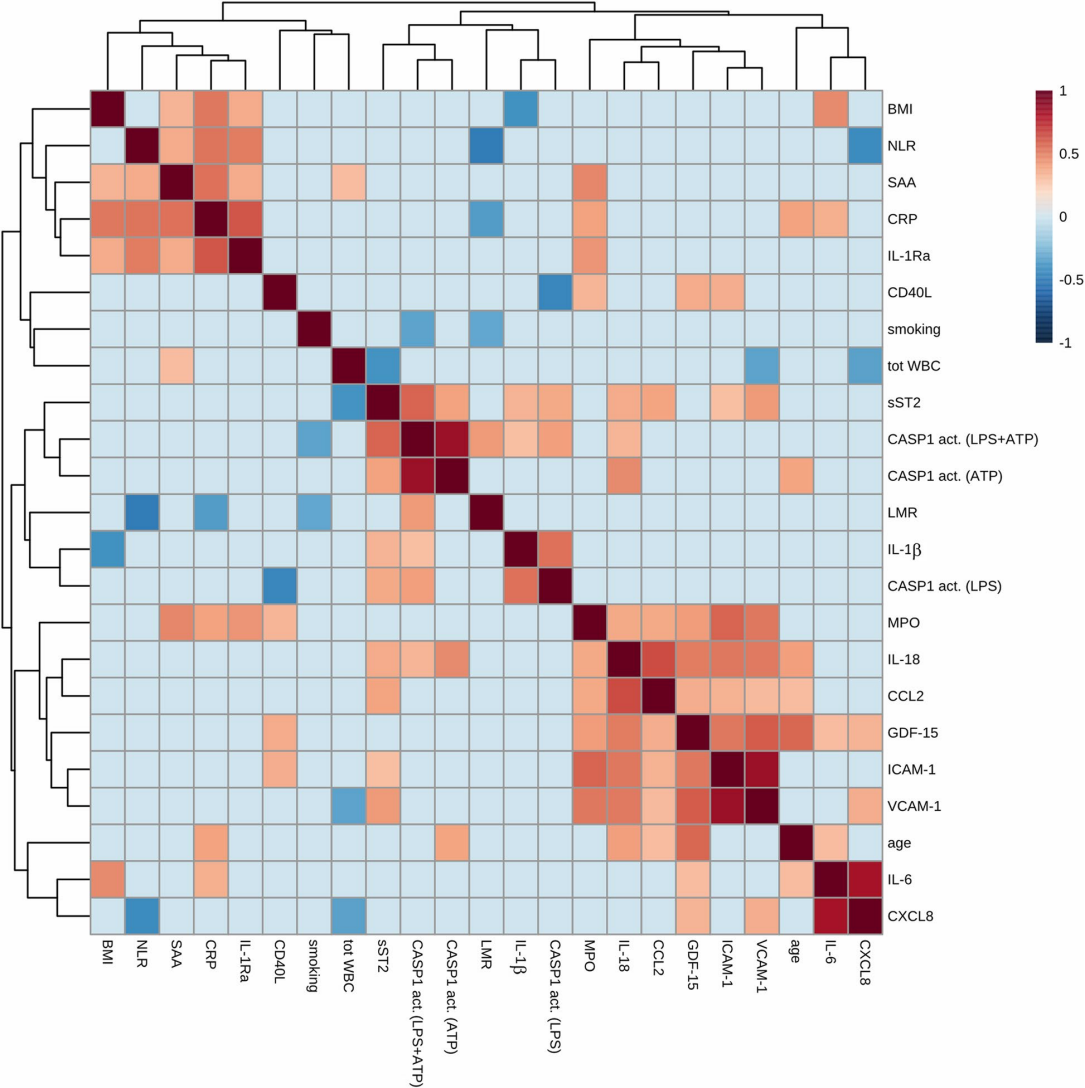
Pearson correlation heatmap of the inflammatory markers of the study using the average morning sample values (*n* = 40) and selected individual properties (BMI, age, smoking). Correlations coefficients < 0.3 are not visualized. All but six of the correlations with a coefficient > 0.3 had a *p*-value < 0.05. The six features > 0.05 all had *p*-values between 0.05 and 0.06. Box Cox transformation was applied to normalize the distribution of the data. White blood cell counts were excluded to improve readability of the figure. NLR: neutrophil–lymphocyte ratio, LMR: lymphocyte-monocyte ratio, casp1 act.: caspase-1 activation of monocytes ex vivo following indicated activators; LPS: lipopolysaccharide, ATP: adenosine triphosphate

A correlation heatmap using Pearson correlation of: 1) the average morning levels of the studied inflammatory markers for each study participant, and 2) selected individual properties (age, BMI, smoking) is shown in Fig. 4. From the heatmap, two distinct clustered blocks appear. The first block includes general markers of inflammation that are correlated with each other; the acute phase proteins CRP and SAA, the cytokine IL1-Ra, and the cell ratio of NLR. In addition, BMI was correlated with most of the above-mentioned markers. The other cluster with correlated markers included MPO, IL-18, CCL2, GDF-15, ICAM-1, and VCAM-1. Furthermore, many of the NLRP3 inflammasome related markers did correlate, including plasma levels of the inflammasome-released cytokines IL-1β and IL-18 with most of the caspase-1 enzymatic activity measures in monocytes from ex vivo inflammasome stimulated whole blood. These correlations were most pronounced for IL-1β with LPS activation and IL-18 with ATP activation of caspase-1. sST2, belonging to the IL-1 receptor family, also correlated with the caspase-1 measures, and in addition, with IL-1β as well as IL-18.

### Correlation of inflammatory markers to particle exposure

The inflammatory markers significantly correlated with respirable dust and respirable quartz exposure, respectively, using Pearson’s r and Kendall $\uptau$, are shown in Table 4. The over-shift difference in inflammatory marker levels was used as a measure of acute effects of exposure from that workday. In these analyses, the inflammatory marker and exposure data for each sampling occasion were used (*n* = 69). In addition, the morning pre-shift samples were used to evaluate effects of exposure from recent days/weeks, with the assumption that the averaged exposure for the two sampling occasions for each worker reflects the average daily exposure levels for the time-period between the first and second measurement. The average exposure levels for each worker were analyzed with the morning pre-shift inflammatory marker levels of the follow-up measurement (*n* = 32), or the average morning inflammatory marker levels of the two separate measures (*n* = 40).

**Table 4:** Correlation of inflammatory markers with exposure using Pearson’s r or Kendall $\uptau$ statistics

Inflammatory markers	Sample data	Exposure	Pearson's r		Kendall $\uptau$	
**Caspase-1 e*x vivo* activation in monocytes**			r	*P*-value	$\uptau$	*P*-value
Caspase-1, LPS activated	Morning $\overline{\times }$ 1^st^, 2^nd^	Resp. dust	-0.55	2.65 × 10^–4^	-0.34	0.0021
	Morning 2^nd^	Resp. dust	-0.60	2.56 × 10^–4^	-0.43	6.20 × 10^–4^
	Morning 2^nd^	Resp. quartz			-0.40	0.0016
Caspase-1, ATP activated	Morning $\overline{\times }$ 1^st^, 2^nd^	Resp. quartz	-0.41	0.0089		
	Morning 2^nd^	Resp. quartz	-0.59	4.35 × 10^–4^		
	Shift difference	Resp. dust	0.40	7.69 × 10^–4^	0.28	0.0011
	Shift difference	Resp. quartz	0.39	0.0012	0.26	0.0027
Caspase-1, LPS + ATP activated	Morning $\overline{\times }$ 1^st^, 2^nd^	Resp. quartz	-0.48	0.0019	-0.30	0.0075
	Morning 2^nd^	Resp. quartz	-0.66	3.31 × 10^–5^	-0.45	2.98 × 10^–4^
**Inflammatory mediators**						
CXCL8	Morning 2^nd^	Resp. dust	0.36	0.0045		
SAA	Shift difference	Resp. dust			-0.22	0.0087
	Shift difference	Resp. quartz			-0.27	0.0014
sST2	Morning 2^nd^	Resp. quartz	-0.55	0.0010	-0.38	0.0022

The inflammatory markers with a significant correlation to respirable dust and/or quartz are shown (*p* < 0.01). The full list of studied inflammatory markers is found in Fig. 3. Over-shift differences in inflammatory markers are used as a measure of effects from current days exposure, whereas the morning samples are used as a measure of effects from recent days/weeks of exposure, assuming similar exposures as measured for the time period between the first and second measurement. Morning $\overline{\times }$ 1^st^, 2^nd^: the average inflammatory marker and exposure level from the two sampling occasions for each participant. Morning 2^nd^: The morning inflammatory marker levels of the second measurement (follow-up measurement) correlated to the averaged exposure level for the two sampling measures

*LPS* Lipopolysaccharide, *ATP* Adenosine triphosphate, *resp.* Respirable

Regarding acute over-shift effects, caspase-1 activity in monocytes following ex vivo ATP treatment correlated with both respirable dust and respirable quartz levels. In addition, there was a negative correlation between SAA and both exposure measures.

For the morning samples indicating effects from recent days/weeks exposure, the ex vivo inflammasome activation assays had a significant negative correlation to exposure. The respirable dust and respirable quartz exposure was negatively correlated with caspase-1 activation following LPS-treatment ex vivo, whereas the respirable quartz exposure had a negative correlation with caspase-1 activation following ATP or LPS + ATP ex vivo treatment. sST2 and CXCL8 were the only non- inflammasome related inflammatory markers that significantly correlated with exposure for the morning samples; sST2 showing a negative correlation with quartz and CXCL8 a positive correlation with respirable dust exposure.

**Fig. 5:**
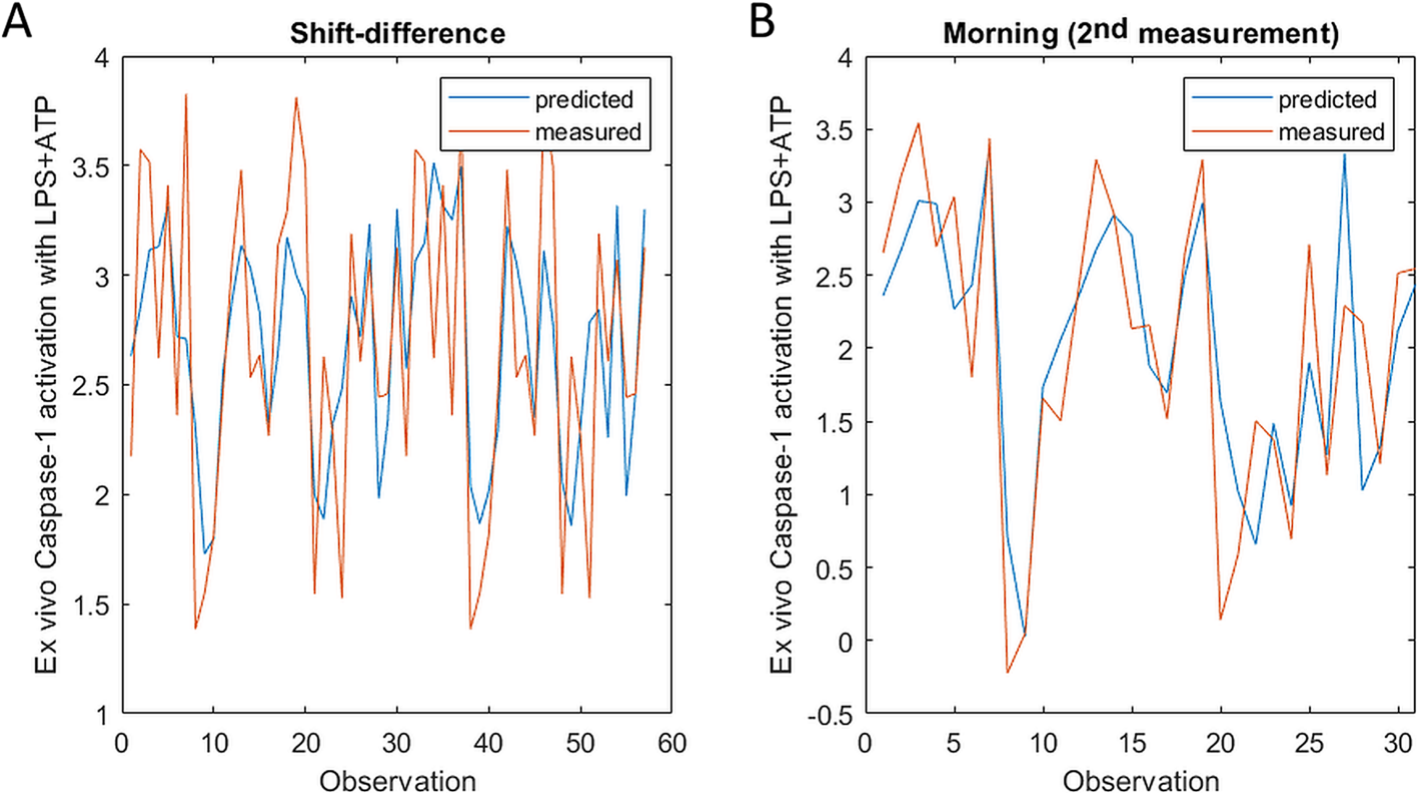
Visualizing the fit of the mixed model correlating quartz exposure with over-shift differences (**A**) and morning levels (**B**) of caspase-1 ex vivo activation with LPS + ATP in monocytes from participating foundry workers (*n* = 69 measurements from 40 individuals for the over-shift difference (**A**), and *n* = 32 for the morning, 2^nd^ measurement (**B**)). The parameters quartz exposure, age, BMI, and smoking were included in model (**A**), and in addition workplace (**B**) determined by the Akaike information criterion. Caspase-1 enzymatic activity was stimulated by treatment of the blood samples with 10 pg/mL LPS for 3 h, followed by 1 h treatment together with 250 µM ATP and the FAM-YVAD-FMK caspase-1 enzymatic activity probe, before determination of the percent caspase-1 activated monocytes. The figure displays the Box Cox transformed data. LPS: lipopolysaccharide, ATP: adenosine triphosphate

To evaluate the impact of covariates, such as age, smoking and BMI, a mixed model analysis was performed on the inflammasome-related markers as well as on other markers that were found to have significant correlation to quartz or dust exposure using Pearson or Kendall $\uptau$ correlation, i.e., CXCL8, SAA and sST2. In the mixed models, shown in Table 5, the over-shift difference and morning sample analyses gave similar results regarding what markers significantly correlated to exposures, resembling the results for the morning values using Pearson correlation. For both the over-shift and morning measures, ex vivo caspase-1 activation following ATP treatments alone or in combination with LPS were found to significantly negative correlate to quartz exposure. LPS treatment alone also correlated negatively to respirable dust exposure; however, only significantly for the over-shift difference. Compared to the Pearson analysis, there was a negative correlation to quartz exposure for some additional inflammasome markers including IL-1β (over-shift difference) and IL-18 (morning levels and over-shift difference). In addition, respirable dust exposure positively correlated to the inflammatory markers CXCL8 (morning samples), and sST2 (over-shift difference), and sST2 additionally correlated negatively with quartz exposure. Additional statistics for the mixed models are shown in Additional file 1: Table S2 and Table S3. The mixed models of caspase-1 activation by LPS + ATP demonstrated the best fit to the observed data, determined by the adjusted R^2^ value and by visual inspection (Fig. 5).

**Table 5:** Mixed model analysis on the effect of respiratory dust or quartz exposure on selected inflammatory markers

Inflammatory marker	Sample data	Exposure	*P* value	R^2^ adj	Direction	Other variables included
**Inflammasome-related cytokines**						
IL-1β	Morning	Quartz	0.15	0.047	Down	Infection
	Shift difference	Quartz	0.0039	0.25	Down	Age, workplace, smoking, stress
IL-18	Shift difference	Quartz	0.014	0.29	Down	Age
	Morning	Quartz	0.049	0.32	Down	Age, BMI, stress, infection
IL-1ra		N/S by AIC				
**Caspase-1 e*x vivo* activation in monocytes**						
Caspase-1 LPS act	Morning	Resp. dust	0.052	0.54	Down	Workplace, stress infection
	Shift difference	Resp. dust	0.0036	0.24	Down	BMI, workplace, stress
Caspase-1 ATP act	Morning	Quartz	2.76 × 10^–5^	0.47	Down	Age, BMI
	Shift difference	Quartz, Resp. dust	0.0028 0.16	0.44	Down Up	Pre-shift value, age, BMI, workplace
Caspase-1 LPS + ATP act	Morning	Quartz	1.90 × 10^–6^	0.61	Down	Age, BMI, workplace, smoking
	Shift difference	Quartz	9.63 × 10^–5^	0.45	Down	Age, BMI, smoking
**Other inflammatory mediators significantly correlated with exposure using Pearson or Kendall** ${\varvec{\uptau}}$						
CXCL8	Shift difference	Resp. dust	0.060	0.15	Up	BMI
	Morning	Resp. dust	0.028	0.20	Up	BMI
SAA		N/S by AIC				
sST2	Morning	Quartz	0.0047	0.27	Down	Workplace
	Shift difference	Quartz	0.020	0.13	Down	BMI, workplace
		Resp. dust	0.029		Up	

Mixed model analysis performed on inflammasome-related markers and inflammatory markers which significantly correlated with exposure using Pearson or Kendall $\uptau$. The Akaike information criteria was used to select models, by testing the variables respiratory quartz exposure, respiratory dust exposure, BMI, age, smoking status, symptoms of infection last two weeks, mental stress, and workplace (foundry A or B). For the over-shift difference data, the pre-shift inflammatory marker levels were also included as a variable. For the morning samples, the inflammatory marker data from the second sampling and the average exposure for each individual was included in the analyses. For the over-shift differences, the biomarker data and exposure data from both sampling occasions were included in the analyses. The R^2^ adj. value was adjusted for the number of parameters included in the model

*N/S* Not selected, *act.* Activated, *resp.* Respirable

## Discussion

Respirable dust and quartz exposure in the iron foundry environment is a potential health hazard that, in accordance with other particle exposure, e.g., in the ambient environment, could mediate inflammatory reactions contributing to the development of e.g., CVD. Therefore, in this study, the dust and quartz exposure, respectively, were characterized in two Swedish iron foundries and their effects on markers of inflammation were studied, including: 1) general markers of inflammation that included the acute phase proteins CRP and SAA, leukocyte cell counts, such as total WBC and the cell ratios NLR and LMR, and the cytokines/chemokines IL-6, CXCL8 and CCL2, 2) established and novel potential CVD biomarkers, including ICAM-1 [20], VCAM-1 [21], GDF-15 [22], MPO [23], sST2 [24], and CD40L [25], and 3) NLRP3 inflammasome associated markers, including IL-1β, IL-18, IL-1Ra, and inflammasome activation to ex vivo activating stimuli, measured by caspase-1 enzymatic activity in monocytes.

Previous studies, using lag days of exposure, have found that PM exposures can affect different inflammatory markers either very fast, with lag 0–24 h [26, 27], or more slowly, with the largest effects after 24–95 h post exposure [26], or from the average 5-day lag measure [26, 28]. In the present study, the over-shift variation in inflammatory marker levels were analyzed in regard to exposure to find effects on fast inflammatory responses to exposure. Slower, retained inflammatory responses from recent days/weeks of exposure were studied by correlating the average exposure for the two exposure measures with either i) the morning pre-shift inflammatory marker levels for the follow-up measurement or ii) the average morning inflammatory marker levels from the two measures. Although large temporal variability in exposure is possible for single days, the correlation across the repeated exposure measurements was significant, indicating similar daily exposures for this time-period for most workers. During the time between the two campaigns, three workers reported a change in work tasks and three workers reported days of absence due to childcare or sick leave. Thus, the majority of workers were fixed to their work tasks, data that strengthen the study design.

The average respirable dust and quartz exposures (1.3 and 0.065 µg/m^3^, respectively) were similar to our previous measures in a Swedish iron foundry cohort (0.85 and 0.052 mg/m^3^, respectively) [29]. When adjusted for respirator use, no measurement exceeded the current Swedish OEL of 2.5 and 0.1 mg/m^3^, for respirable dust (average 0.65 mg/m^3^) and quartz (average 0.020 mg/m^3^), respectively. However, in six out of 72 measurements, the levels of respirable quartz were above 0.5 mg/m^3^, demonstrating exposure levels close to OELs in some parts of the production line. To further characterize the foundry dust, we measured the size distribution of the PM_2.5_, composed of particles roughly comparable in size to the respirable fraction that collects PM < 4 µm. As shown by the stationary measurements of respirable dust and PM_2.5_ in Table 1, the average exposure to these two size fractions in the foundries were at similar levels. Interestingly, ultrafine/nanoparticles (< 0.25 µm) constituted a large mass portion (37%) of the PM_2.5_. Such small nano-particles may have the ability to cross the lung-epithelial barrier, thereby gaining access to the circulatory system where they may induce direct effects on other parts of the body, including cardiovascular tissues [2]. On the other hand, our results show that the quartz content is reduced in the fractions of smaller particles, going from 3.7% in the > 2.5 µm fraction down to 1.1% in the < 0.25 µm fraction. Thus, the majority of quartz particles reaching the lower parts of the lungs are likely in the µm range and retained in that part of the lung. By visual observation of the collected particles, the smallest particles (< 0.25 µm) appear, however, to be constituted by a larger proportion of metal content, such as iron oxides, as indicated by the red/brown color observed in the majority of those dust samples. Effects of exposure to iron oxides of low µm-sized particles from occupational exposure or in controlled human exposure chamber studies generally show low toxicity [30]; however, the toxicity of iron oxide particles in the nano-sized range needs further investigation.

Further, the metal content of collected inhalable dust filters was analyzed, revealing large variations in the metal content in regard to different sampling occasions and locations in the foundries, ranging from 5–45% of the total mass. The most abundantly identified metal was iron, followed by magnesium and aluminum. The fettling area was the only location that had particle levels for metals (*i.e.*, iron, copper, chromium) most likely above the Swedish OELs, given that the OELs was set for a different size fraction. However, the workers participating in the current study always wore protective equipment when working at this particular workplace. The large variation in the composition of the dust may complicate the use of general classification of dust fractions, such as respirable dust, when correlating inflammatory markers with exposure. In particular acute effects are difficult to evaluate given the fact that a certain fraction of the dust particles within the mix may exert the majority of the biological responses.

Effects of short-term exposure have been shown for ambient particles, including effects on inflammation [13, 14], likely contributing to increased mortality and morbidity in CVDs associated with acute exposure [31]. In the current study, effects of exposure to respirable quartz and respirable dust were examined by using the over-shift differences or morning pre-shift levels in inflammatory markers. Some of the inflammatory markers investigated in the current study had significantly different levels in the morning and afternoon samples without displaying significant correlation to exposure; data indicating diurnal variations, with examples of both significantly higher levels in the afternoon (*e.g.*, neutrophils and MPO) and lower levels (*e.g.*, CCL2 and CRP). Other markers did not display any significant diurnal variation, including the adhesion molecules ICAM-1 and VCAM-1 as well as the cytokines IL-1β and GDF-15.

There was a significant correlation between some of the over-shift differences in inflammatory marker levels and exposure, including respirable dust and respirable quartz exposure with SAA and ex vivo ATP-induced caspase-1 activation. SAA is an acute phase protein and a sensitive marker of inflammation induced by e.g., infection, injury or stress, and elevated levels have been associated with adverse cardiovascular outcomes [32, 33]. In our previous study, SAA was found to correlate with dust exposure in foundry workers [29]. Increased levels of SAA have also been found in the biological response to acute exposure to ambient ultrafine particulate matter [13]. However, regarding SAA in the current study, when adjusting for covariates in the mixed models, no exposure measures were selected as variables by the Akaike information criterion, suggesting a minor role of exposure on SAA levels compared to variables like BMI and symptoms of infection. Instead, when adjusting for covariates, several inflammasome-related markers were found to correlate to respirable quartz, including IL-1β, IL-18, and ex vivo caspase-1 activity. In addition, the inflammatory markers sST2 correlated with quartz and respirable dust exposure. Most markers had a negative correlation to exposure, indicating either a smaller increase or a larger decrease in the post-shift levels compared to the basal morning levels.sST2, a receptor of the IL-1 receptor family binding the ligand IL-33, is suggested to have prognostic and diagnostic value in CVDs, especially for heart failure [34, 35]. In addition, elevated sST2 levels has been reported in different pulmonary diseases and to give prognostic value of mortality in these diseases [36, 37]. There are only a few studies that have examined sST2 in the context of PM exposure; one demonstrating elevated sST2 serum levels in non-farmers exposed to organic dust in a pig barn [38], and one study demonstrating elevated sST2 mRNA levels in lung homogenates in diesel exhaust particles plus house mite dust exposed mice [39].

Effects of dust or quartz exposure on inflammation were also assessed by the morning pre-shift inflammatory marker levels, indicating a more sustained low-grade inflammatory effect of recent days/weeks of exposures. The morning levels were mainly correlated with exposure for inflammasome-related markers, i.e., all of the ex vivo caspase-1 activity measures, and when adjusting for covariates, also IL-18, showing a negative correlation to exposure. In addition, the morning levels correlated with respirable dust and quartz exposure for the inflammatory markers CXCL8 and sST2, respectively, in line with results for the over-shift difference. CXCL8 (interleukin-8) is a chemoattractant for neutrophils that has been found to be produced in response to PM exposure, e.g., in bronchial wash fluid following diesel exhaust particles (DEP) [40], and to be produced by lung epithelial cells following DEP and silica exposure [41].

When adjusting for covariates, both the morning levels and the over-shift difference had very similar results, indicating correlation to quartz exposure for the inflammasome-related markers IL-18 and ex vivo caspase-1 activation. In a previous study of 85 foundry workers, similar results were found, showing correlation between respirable dust and quartz and effects on inflammasome/caspase-1 activation, and in addition correlation of IL-18 and IL-1Ra with dust exposure [42]. Taken together, these results indicate that the dust and quartz exposure in the iron foundry environment affect the NLRP3 inflammasome/caspase-1 axis. The inflammasome generally require two signals to be assembled, leading to activation of pro-caspase-1 into active caspase-1 that in turn cleaves IL-1β and IL-18 into their active forms. In our ex vivo inflammasome activation experiments, LPS was used as the first “priming” signal that induces transcription of NLRP3 inflammasome components, including NLRP3 and the pro-forms of the cytokines IL-1β and IL-18. Further, ATP was used as the secondary “triggering” signal that govern the assembly of the components caspase-1, NLRP3, and ASC into an active inflammasome. Interestingly, respirable dust exposure correlated with caspase-1 effects by LPS treatment, indicating an effect on inflammasome priming, whereas the respirable quartz exposure correlated to caspase-1 effects by ATP treatments, indicating effects on the secondary inflammasome assembly signal and that the monocytes extracted from foundry workers were not naïve but predisposed (primed) for inflammasome activation. Quartz is a well-known activator of the inflammasome [43], and these results suggest that quartz exposure has a biological effect that was induced also at the current exposure levels detected in the foundries of the study, i.e., below the OEL. The inflammasome is suggested to play an important role in particle/quartz mediated diseases, including CVD [44] and lung diseases, such as silicosis and chronic obstructive pulmonary disease [43, 45]. Therefore, finding exposure levels that do not affect inflammasome signaling would be desirable in order to prevent biological impact before turning into disease.

There are some limitations of the study, mainly the relatively low number of participants. This limitation was dealt with using repeated measures to give high quality of the exposure and biomarker data. The diurnal variation can be viewed as a problematic issue when comparing biomarker levels at different time points. However, observing a reduced or increased change in the diurnal variation could also be an important aspect of the inflammatory response, rather than considering merely increased levels of inflammatory markers post-shift as signs of inflammation. Finally, acute effects on systemic inflammation may take longer than one day to wash-out. For example, a study on ambient air pollution and the effects on inflammatory/coagulation markers in susceptible individuals demonstrated the strongest correlations for PM_2.5_ with CRP for a lag time of 24–95 h post exposure, or the 5-day lag average exposure, while other markers, including sCD40L and PAI-1 demonstrated significant correlation with PM_2.5_ only for the 0–23 h lag time [26]. Thus, it seems that inflammatory markers vary in their response times to exposure, and that there is likely no way to get around this, e.g., by sampling on a Monday. Therefore, we chose a workday in the middle of the workweek rather than a Monday for blood sampling and exposure measurement.

## Conclusions

The results demonstrate that exposure to respirable dust and quartz in the iron foundry environment correlate with systemic inflammatory markers, including effects on inflammasome activation and with the inflammatory markers sST2, and CXCL8. The exposure levels were, when corrected for respirator use, below the Swedish OELs. Still, as correlation of exposure to biological effects were detected, concerns must be raised about the safety of the current exposure levels, and iron foundries and industries of similar exposures are therefore encouraged to continuously work towards reduced dust and quartz exposures in the workplace.

## Methods

### Study group

The study was performed on 40 individuals working at two Swedish iron foundries, employing in total ca 90 and 25 individuals, respectively. One foundry manufactures parts for wind turbines and the other manufacture mainly custom orders. The produced castings at both sites are mainly comprised of iron and grey iron alloys. Descriptive statistics of the study population and their employment can be seen in Table 6. The participants were employed for work tasks throughout the production chain, including mainly work in generating the casted goods (mold making, core making, melting, casting, shake out, and fettling). Additional work included product controller, truck driver, feedstock work, maintenance, and administrative/leadership work. Exclusion criteria included pregnancy and diabetes.

**Table 6:** Descriptive statistics of the 40 iron foundry workers included in the study

Gender	Male	Female		
	38	2		
Age	Mean	SD	Min	Max
	42	11	20	63
BMI	Mean	SD	Min	Max
	29	5	20	41
Employment time (years)	Mean	SD	Min	Max
	9	7	0	27
Smoking	Current smoker	Ex-smoker	Never-smoker	
	10	11	19	
Workplace	Factory A	Factory B		
	20	20		
Main worktask (*n* = 72 measurements)	Casting/molding	Core Making	Melting	Fettling
	19	9	12	10
	Shake out	Maintenance/ quality control	Other work in production	Administrative work
	3	9	3	7

### Study design

**Fig. 6:**
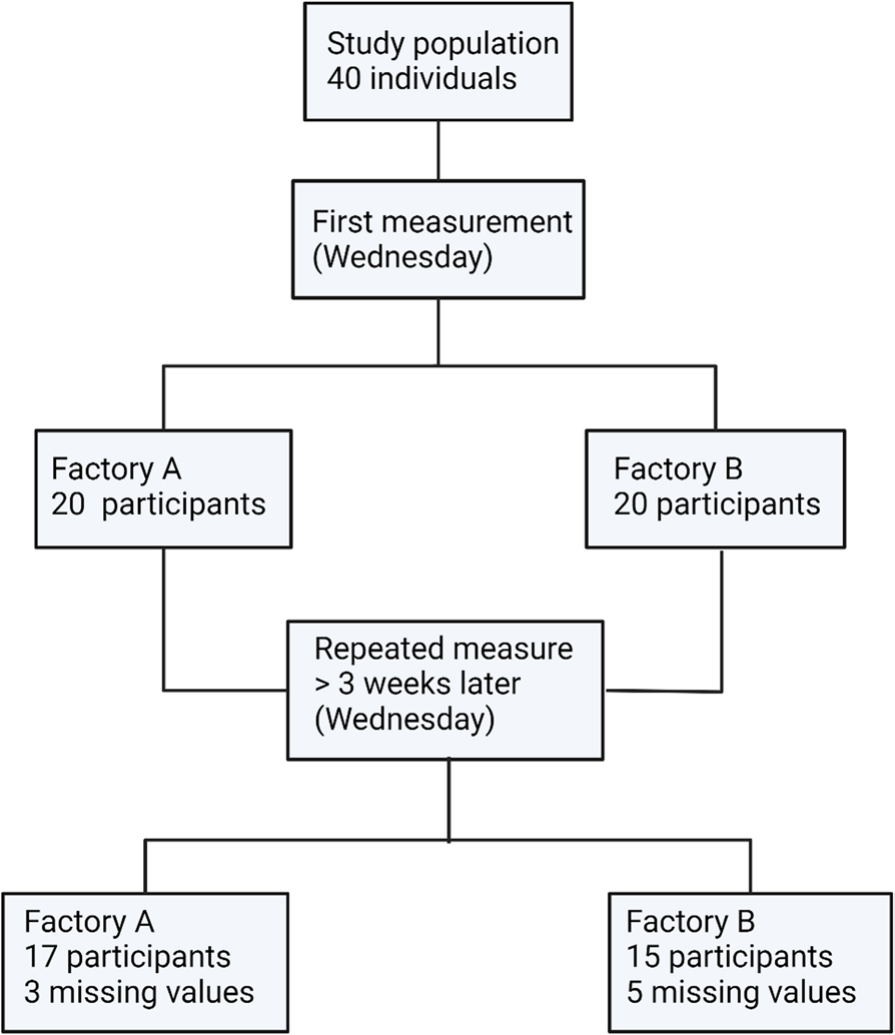
Schematic illustration of the sampling procedure

The inclusion of the study was performed between January 2019 and February 2020 at two different Swedish iron foundries during eight different sampling occasions, sampling *ca* 10 individuals per campaign. At the smaller foundry, all participants, who met the inclusion criteria were offered to participate, and at the larger foundry, all foundry workers working the dayshift at the sampling dates were offered to participate. A schematic illustration of the sampling procedure is shown in Fig. 6. Sampling was done during the months October – March to avoid the pollen season. Air sampling of dust levels was performed during the third day following a work-free weekend. On the same day, venous blood was collected in the morning before work (pre-shift), and in the afternoon after an eight-hour work-shift (post-shift). The morning samples were overnight fasting samples. Repeated measures were conducted on the same individuals at least 3 weeks after the first measurements. On the repeated measurement, it was not possible to sample 8 of the individuals as they were absent from work on the sampling occasion, due to sick leave, childcare, or work travel. To generate blood plasma, the tubes with collected venous blood were immediately put on ice and were within 30 min centrifuged at 2 000 × g for 15 min. Li-heparin plasma was refrigerated until analysis of CRP, whereas the EDTA-plasma was frozen on site and then stored in the biobank until subjected to biomarker analysis. In addition, on-site measurement of white blood cell (WBC) counts and ex vivo stimulation of whole blood for assessment of inflammasome activation was performed. A questionnaire was completed by all participants, gathering information about working conditions, age, sex, body mass index (BMI), and smoking habits.

### Aerosol measurements and characterization of quartz and metal content

Measurements of respirable dust and quartz were carried out as personal sampling for all study participants, using a SKC aluminium cyclone (SKC 225–01-01, SKC, Eighty Four, PA) equipped with a nitrocellulose membrane filter and an air pump (SKC AirCheck 5 000) operating at a constant airflow rate of 2.5 L/min. The workers received the sampling equipment before work started and they carried the equipment throughout their eight-hour work-shift. In addition, stationary measurements were performed for additional particle measures, including inhalable dust, respirable dust, PM_10_, PM_2.5_, PM_1_, particle number concentration, and particle surface area concentration. Also, a Sioutas cascade impactor (SKC) was used to measure dust and quartz levels at five cut-points (2.5 µm, 1 µm, 0.5 µm, 0.25 µm, and < 0.25 µm aerodynamic diameter). The dust concentrations on the filters were analyzed gravimetrically and the quartz content of collected respirable dust and impactor filters were analyzed by X-ray diffraction on a X’Pert PRO instrument (Malvern Panalytical, Malvern, Worcestershire, United Kingdom) with the angles 20°, 26° and 50°. Prior to analysis, the collected dust filters were ashed using a EMITECH K1050X radio frequency plasma asher (Emitech, Montigny-le-Bretonneux, France) at 60 °C for 14 h, and the remaining inorganic material was wet filtered onto a silver membrane filter (0.8 µm pore size) to be used in the X-ray diffraction analysis. For the inhalable particle fraction, the metal content of the collected dust was analyzed using an iCAP Q ICP-MS instrument (Thermo Fisher Scientific, Waltham, MA) performed after the filters had been dissolved in acid (concentrated nitric acid with 10% hydrogen peroxide).

### Cytokine measurements

The plasma concentration was determined for 16 different proteins, both in the morning (pre-shift) and afternoon (post-shift) samples for the 40 study participants. The proteins measured, and details of the analyses, are shown in Table 7. High sensitivity CRP was analyzed at the Department of Clinical Chemistry, Örebro University Hospital, and IL-1β was analyzed by Quanterix (Billerica, MA) sample analysis service at the company’s facility. All other protein biomarkers were analyzed by Mesoscale Diagnostics (Rockville, MD) technology at Örebro University according to the manufacturer’s instructions, with technical duplicates, and signals were detected using the QuickPlex SQ120 instrument (Mesoscale diagnostics).

**Table 7:** Inflammatory proteins measured in blood plasma

Analyte	Abbreviation	Sample type	Analysis method
**General inflammatory proteins**			
C-reactive protein	CRP	Li-heparin plasma	High sensitivity
C-X-C Motif Chemokine Ligand 8	CXCL8, (IL-8)	EDTA plasma	MSD—U-plex
Interleukin 6	IL-6	EDTA plasma	MSD—U-plex
Interleukin 17A	IL-17A	EDTA plasma	MSD—U-plex
Interleukin 33	IL-33	EDTA plasma	MSD—U-plex
C–C motif ligand 2	CCL2 (MCP-1)	EDTA plasma	MSD—U-plex
Serum amyloid A	SAA	EDTA plasma	MSD—V-plex
**Novel inflammatory CVD markers**	ICAM-1	EDTA plasma	MSD—V-plex
Intercellular adhesion molecule 1	GDF15	EDTA plasma	MSD—R-plex
Vascular cell adhesion molecule 1	VCAM-1	EDTA plasma	MSD—V-plex
CD40 ligand	CD40L	EDTA plasma	MSD—R-plex
Growth/differentiation factor 15	GDF-15	EDTA plasma	
Myeloperoxidase	MPO	EDTA plasma	MSD—R-plex
Soluble suppression of tumorigenesis 2	sST2	EDTA plasma	MSD—R-plex
**NLRP3 inflammasome related proteins**			
Interleukin 1 beta	IL-1β	EDTA plasma	Simoa bead tech
Interleukin 1 receptor antagonist	IL-1Ra	EDTA plasma	MSD—U-plex
Interleukin 18	IL-18	EDTA plasma	MSD—U-plex

*EDTA* Ethylenediaminetetraacetic acid, *MSD* Mesoscale Diagnostics

### White blood cell counts

Cell counts were performed in freshly isolated blood for total WBCs, neutrophils, lymphocytes, monocytes, eosinophils, and basophils using the Hemocue WBC Diff instrument (Hemocue, Ängelholm, Sweden). From these data, the neutrophil-to-lymphocyte ratio (NLR) and lymphocyte-to-monocyte ratio (LMR) was calculated.

### Ex vivo NLRP3 inflammasome activation

**Fig. 7:**
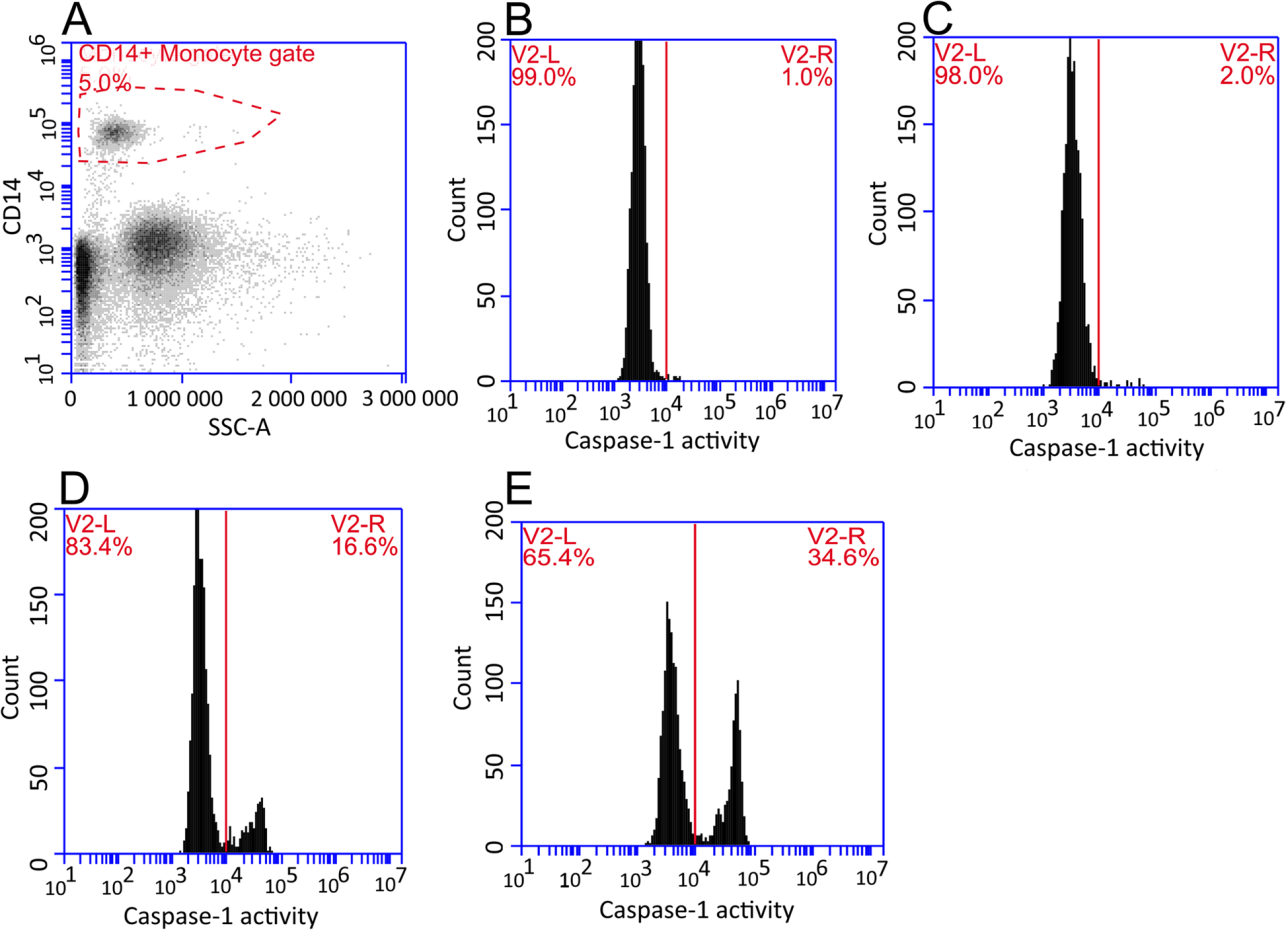
Representative flow cytometry plots used to determine the percent of monocytes in the caspase-1 positive (inflammasome activated) peak. **A** Gating of CD14^+^ monocytes. **B**-**E** Caspase-1 enzymatic activity in gated monocytes measured by the FAM-YVAD-FMK probe in **B** untreated cells, **C** LPS treated cells, **D** ATP treated cells, **E** LPS + ATP treated cells. V2-R indicates caspase-1 (inflammasome) activated cells

For analysis of caspase-1 activity in circulating monocytes, 100 µL freshly isolated whole blood (EDTA treated, used within 3 h after sampling) from the study participants was mixed with an equal volume of RPMI 1640 cell culture media (Thermo Fisher Scientific, Waltham, MA), either with or without lipopolysaccharide (LPS (ultrapure LPS-B5, Invivogen, San Diego, CA), resulting in 10 pg/mL final LPS concentration. The samples were incubated at 37 °C for three hours before addition of 4.5 µL FAM-FLICA-YVAD-Caspase-1 probe (Immunochemistry Technologies, Bloomington, MN), and 1 µL CD14-APC antibody (IM2580, Beckman Coulter, Brea, CA) or isotype control. In addition, some samples received 250 µM adenosine triphosphate (ATP), resulting in 6 different sample treatments per individual: 1) CD14 isotype control, 2) autofluorescence control (no caspase-1 probe), and the following (samples 3-6) with CD14-APC antibody and caspase-1 probe: 3) non-treated sample, 4) LPS treated sample, 5) ATP treated sample, and 6) LPS + ATP treated sample. The samples were incubated at 37 °C for 1 h prior to fixation and lysis of red blood cells with 1.7 mL eBioscience 1-Step Fix/Lyse Solution (Thermo Fisher Scientific). Following lysis for 15 min, the samples were washed twice with FLICA wash buffer prior to analysis of caspase-1 enzymatic activity by flow cytometry using an Accuri C6 instrument (BD, Franklin Lakes, NJ). In the flow cytometer analysis, monocytes were gated from a CD14 and side scatter plot, and 2 000 gated events were collected for data analysis of caspase-1 activity. Representative plots are shown in Fig. 7.

### Exposure measures

For all participants, 8-h time-weighted average (TWA) exposures were calculated for the personal respirable dust and quartz measurements. Out of the 40 individuals, sampled up to two times (in total 72 personal measurements), respirators were used by 10 individuals (15 measurements) for some part of the day. Therefore, both the respirator-adjusted exposure levels and unadjusted exposure levels were calculated. For 9 of the 15 measurements where respirators were used, a full facepiece with external air supply was used, and for the remaining 6 measurements a half facepiece equipment was used. For the measures where a half facepiece was used, the time spent with respirators were less than half of the workday. In contrast, workers using a full facepiece reported using the respirators for a majority of the time. To calculate respirator-adjusted exposures, zero exposure was assumed during the time a respirator was worn, and during the time without respirator, the exposure was calculated from the background exposure measured by a stationary measurement performed in the proximity of the worker. Since respirators were most likely used during operations with peak exposures, we find the use of background exposures for the time-points without respirator use most accurate. However, if no stationary measurement was considered relevant for an individual, the average personal exposure levels were instead used for the time-points without respirator use.

### Statistical analysis

For analyzing the short-term acute effects of exposure, the post-shift, pre-shift biomarker difference was correlated to exposure. In these analyses, the up to two over-shift biomarker differences and PM exposure levels for each individual were used in the analyses (*n* = 69, for 3 out of the 72 measurements, blood was not retrieved both pre- and post-shift). For analyzing effects of recent days/weeks exposure to respirable dust and quartz with biomarker levels, the average exposure levels for the two repeated measurements were used, with the exception of one individual who permanently changed work tasks from production line to product controller after the first measurement. The exposures were correlated with the morning pre-shift levels, either using the average morning pre-shift levels from the two repeated measures (morning $\overline{\times }$ 1^st^, 2^nd^, *n* = 40), or using the morning samples of the follow-up measurement (morning 2^nd^, *n* = 32). For the morning $\overline{\times }$ measure, the eight individuals that did not come for the follow-up measurement were included. Data were normalized using the Box Cox transformation to approach Gaussian distributions. Log-transformation was selected for all plasma proteins and caspase-1 activity measures. The power transform $\frac{{x}^{\uplambda }-1}{\uplambda }$ was selected for exposure measures ($\uplambda$= 0.2235 and 0.1408 for respirable dust and respirable quartz, respectively), age ($\uplambda$= 1.3822 and BMI ($\uplambda$= -1.8007). For WBC measures and smoking, no transformation was performed due to these parameters either containing zero or negative values or being categorical. Correlation of exposure with normalized biomarker levels were calculated using both ordinary Pearson correlation and Kendall $\uptau$ rank correlation, with a *p*-value threshold of 0.01 for significance. The low *p*-value was chosen to decrease the chance of type I errors (false positives) due to the large number of comparisons. To compare if the over-shift biomarker levels differed significantly, *p*-values were calculated using the Wilcoxon signed rank test. To visualize correlation of biomarker levels, exposures, and individual features (age, BMI, smoking (never/Ex/current smoker)), a heatmap was created from the Pearson correlation of the average morning biomarker levels.

For the mixed model used to model exposure with the over-shift difference in biomarker levels, the following model was considered:

$$Y={C}_{0}+{C}_{1}{X}_{1}+{C}_{2}{X}_{2}+{C}_{3}{X}_{3}+{C}_{4}{X}_{4}+{C}_{5}{X}_{5}+{C}_{6}{X}_{6}+{C}_{7}{X}_{7},$$

$Y$ is the over-shift difference in biomarker level (post-shift – pre-shift value), ${C}_{0},\dots {C}_{7}$ the estimated parameters, and ${X}_{1},\dots {X}_{7}$ the predictors considered (morning value of estimated parameter, respirable dust exposure, respirable quartz exposure, age, BMI, symptoms of infection last two weeks (0,1), mental stress (0–5), workplace (0,1) or smoking (never/ex/current)).

In order to only select the relevant predictors and to avoid overfitting, all possible subsets of these predictors was tested and the best one according to the Akaike information criterion was selected (effectively setting ${C}_{i}=0$ for any unselected predictors).

The statistics were calculated, and Fig. 5 generated, using MATLAB v. R2019 (MathWorks, Natick, MA). Graphs in Fig. 1 and 3 were generated using GraphPad Prism v. 5.03 (GraphPad Software, San Diego, CA), Fig. 4 was generated using the MetaboAnalyst software v.5.0 (www.metaboanalyst.ca). Figure 7 was generated using the BD Accuri C6 software v. 1.0.264.21 (BD).

## Supplementary Information

**Additional file 1: Table S1.** Biomarkers included in the study, with the morning (pre-shift) and afternoon (post-shift) levels shown (*n*= 40 foundry workers). If a participant was sampled more than once, the average level for each time-point is shown. In addition, the over-shift difference (post-pre shift levels) of each biomarker is shown. *P*-value for the over-shift difference was calculated with the Wilcoxon signed-rank test. * Below detection limit for most of the measurements. **Table S2.** Mixed model data for the over-shift difference of selected inflammatory markers. **Table S3.** Mixed model data of selected inflammatory markers for the morning 2nd measurement biomarker levels.

## Data Availability

The datasets used and/or analyzed during the current study are available from the corresponding author on reasonable request.
