# Supplementary material for: Occupational quartz and particle exposure affect systemic levels of inflammatory markers related to inflammasome activation and cardiovascular disease
Source: Environ Health. 2023 Mar 13;22:25. doi: 10.1186/s12940-023-00980-1 (PMC10009934; doi:10.1186/s12940-023-00980-1)
Supplement: Supplementary file 1 — Additional file 1: Table S1. Biomarkers included in the study, with the morning (pre-shift) and afternoon (post-shift) levels shown (n= 40 foundry workers). If a participant was sampled more than once, the average level for each time-point is shown. In addition, the over-shift difference (post-pre shift levels) of each biomarker is shown. P-value for the over-shift difference was calculated with the Wilcoxon signed-rank test. * Below detection limit for most of the measurements. Table S2. Mixed model data for the over-shift difference of selected inflammatory markers. Table S3. Mixed model data of selected inflammatory markers for the morning 2nd measurement biomarker levels. [file 12940_2023_980_MOESM1_ESM.docx]

**Table S1.** Biomarkers included in the study, with the morning (pre-shift) and afternoon (post-shift) levels shown (n= 40 foundry workers). If a participant was sampled more than once, the average level for each time-point is shown. In addition, the over-shift difference (post-pre shift levels) of each biomarker is shown. P-value for the over-shift difference was calculated with the Wilcoxon signed-rank test. * Below detection limit for most of the measurements.

|  | Median | | Mean | | Min | Max | Mean post-pre shift difference | P value |
| --- | --- | --- | --- | --- | --- | --- | --- | --- |
| **Protein plasma concentration (pg/mL**) | Pre-shift | Post-shift | Pre-shift | Post-shift |  |  |  |  |
| CD40L | 2453 | 2783 | 2581 | 2911 | 539 | 6511 | 330 | 0.19 |
| CRP | 2.41 | 2.24 | 3.20 | 2.93 | 0.16 | 15.97 | -0.26 | 0.006 |
| GDF-15 | 1033 | 1080 | 1156 | 1166 | 348 | 3035 | 10 | 0.74 |
| IL-1β | 0.26 | 0.25 | 0.59 | 0.60 | 0.08 | 4.88 | 0.0028 | 0.34 |
| IL-1Ra | 173 | 196 | 262 | 280 | 82 | 1915 | 18 | 0.001 |
| IL-6 | 0.50 | 0.53 | 0.65 | 0.63 | 0.072 | 2.33 | -0.024 | 0.69 |
| CXCL8 (IL-8) | 1.63 | 1.19 | 2.56 | 2.44 | 0.34 | 7.4 | -0.13 | 0.007 |
| IL-18 | 952 | 973 | 1182 | 1220 | 340 | 3007 | 38 | 0.013 |
| CCL2 (MCP-1) | 221 | 189 | 238 | 202 | 34.3 | 514 | -36 | <0.0001 |
| sST2 | 197 | 354 | 205 | 369 | 49 | 1106 | 164 | <0.0001 |
| **Protein plasma concentration (ng/mL)** |  |  |  |  |  |  |  |  |
| ICAM-1 | 994 | 1038 | 1018 | 1016 | 467 | 1976 | -1.7 | 0.58 |
| VCAM-1 | 1080 | 1059 | 1096 | 1083 | 377 | 2473 | -13 | 0.54 |
| SAA | 2567 | 2038 | 4863 | 3398 | 493 | 61251 | -1465 | <0.0001 |
| MPO | 14.1 | 16.0 | 14.9 | 17.1 | 5.1 | 34.6 | 2.2 | 0.0003 |
| IL-17A * |  |  |  |  |  |  |  |  |
| IL-33 * |  |  |  |  |  |  |  |  |
| **Cell counts (10^9^/L)** |  |  |  |  |  |  |  |  |
| Total WBC | 6.0 | 6.9 | 6.0 | 7.1 | 3.8 | 12.6 | 1.1 | <0.0001 |
| Neutrophils | 3.2 | 4.1 | 3.1 | 4.3 | 1.8 | 8.1 | 1.1 | <0.0001 |
| Lymphocytes | 2.3 | 2.2 | 2.3 | 2.3 | 0.6 | 4.1 | -0.066 | 0.24 |
| Monocytes | 0.3 | 0.4 | 0.3 | 0.4 | 0.1 | 1 | 0.088 | 0.0001 |
| Eosinophils | 0.2 | 0.1 | 0.2 | 0.2 | 0 | 0.75 | -0.040 | 0.0032 |
| **Ratios** |  |  |  |  |  |  |  |  |
| NLR | 1.3 | 1.8 | 1.5 | 2.1 | 0.7 | 7.5 | 0.55 | <0.0001 |
| LMR | 7.5 | 6.0 | 7.9 | 6.0 | 2.4 | 14.5 | -1.89 | <0.0001 |
| **Caspase-1 activity, (% activated monocytes) following *ex-vivo* inflammasome stimulation** |  |  |  |  |  |  |  |  |
| Untreated | 1.5 | 2.7 | 2.1 | 3.0 | 0.4 | 8.6 | 0.9 | <0.0001 |
| LPS 1 ng/ml | 2.3 | 3.9 | 2.8 | 4.1 | 0.5 | 10.6 | 1.3 | 0.0005 |
| ATP 250 µM | 2.5 | 3.3 | 4.0 | 5.7 | 0.6 | 21.8 | 1.7 | <0.0001 |
| LPS+ATP | 7.1 | 13.1 | 9.9 | 16.1 | 1 | 41.3 | 6.1 | <0.0001 |

**Table S2**. Mixed model data for the over-shift difference of selected inflammatory markers.

| **IL-1β** | **Estimate** | | **SE** | | **P value** | | **lower** | | **upper** | |  |
| --- | --- | --- | --- | --- | --- | --- | --- | --- | --- | --- | --- |
| Intercept | -1.30 | | 0.34 | | 0.000 | | -1.99 | | -0.62 | |  |
| Quartz | -0.20 | | 0.065 | | 0.004 | | -0.33 | | -0.067 | |  |
| Age | -0.0015 | | 0.0010 | | 0.136 | | -0.0034 | | 0.00047 | |  |
| Workplace | 0.24 | | 0.092 | | 0.013 | | 0.053 | | 0.42 | |  |
| Smoking | -0.079 | | 0.054 | | 0.148 | | -0.19 | | 0.029 | |  |
| Stress | 0.37 | | 0.12 | | 0.005 | | 0.12 | | 0.61 | |  |
|  |  | |  | |  | |  | |  | |  |
|  |  | |  | |  | | **R^2^** | | **R^2^ adjusted** | |  |
|  |  | |  | |  | | 0.32 | | 0.25 | |  |
|  |  | |  | |  | |  | |  | |  |
| **IL-18** | **Estimate** | | **SE** | | **P value** | | **lower** | | **upper** | |  |
| Intercept | 5.29 | | 0.26 | | 0.000 | | 4.76 | | 5.82 | |  |
| Quartz | -0.17 | | 0.068 | | 0.014 | | -0.31 | | -0.037 | |  |
| Age | 0.0043 | | 0.0010 | | 0.000 | | 0.0024 | | 0.0063 | |  |
|  |  | |  | |  | |  | |  | |  |
|  |  | |  | |  | | **R^2^** | | **R^2^ adjusted** | |  |
|  |  | |  | |  | | 0.32 | | 0.29 | |  |
|  |  | |  | |  | |  | |  | |  |
| **IL-1Ra** | **Estimate** | | **SE** | | **P value** | | **lower** | | **upper** | |  |
| Intercept | -180 | | 53 | | 0.001 | | -286 | | -74 | |  |
| BMI | 334 | | 95 | | 0.001 | | 142 | | 525 | |  |
| Workplace | 0.31 | | 0.093 | | 0.002 | | 0.12 | | 0.49 | |  |
| Smoking | -0.12 | | 0.061 | | 0.046 | | -0.25 | | -0.0026 | |  |
|  |  | |  | |  | |  | |  | |  |
|  |  | |  | |  | | **R^2^** | | **R^2^ adjusted** | |  |
|  |  | |  | |  | | 0.30 | | 0.26 | |  |
|  |  | |  | |  | |  | |  | |  |
| **ATP** | **Estimate** | | **SE** | | **P value** | | **lower** | | **upper** | |  |
| Intercept | 466 | | 102 | | 0.000 | | 261 | | 670 | |  |
| Pre-shift value | -0.20 | | 0.091 | | 0.032 | | -0.39 | | -0.018 | |  |
| Resp. dust | 0.20 | | 0.14 | | 0.157 | | -0.081 | | 0.49 | |  |
| Quartz | -0.56 | | 0.18 | | 0.003 | | -0.92 | | -0.20 | |  |
| Age | 0.0068 | | 0.0019 | | 0.001 | | 0.0029 | | 0.011 | |  |
| BMI | -844 | | 184 | | 0.000 | | -1215 | | -474 | |  |
| workplace | 0.82 | | 0.19 | | 0.000 | | 0.43 | | 1.21 | |  |
|  |  | |  | |  | |  | |  | |  |
|  |  | |  | |  | | **R^2^** | | **R^2^ adjusted** | |  |
|  |  | |  | |  | | 0.50 | | 0.44 | |  |
|  |  | |  | |  | |  | |  | |  |
| **LPS+ATP** | **Estimate** | | **SE** | | **P value** | | **lower** | | **upper** | |  |
| Intercept | 369 | | 79 | | 0.000 | | 211 | | 528 | |  |
| Quartz | -0.43 | | 0.10 | | 0.000 | | -0.63 | | -0.22 | |  |
| Age | 0.0066 | | 0.0014 | | 0.000 | | 0.0037 | | 0.0095 | |  |
| BMI | -665 | | 143 | | 0.000 | | -952 | | -379 | |  |
| smoking | -0.14 | | 0.090 | | 0.131 | | -0.32 | | 0.042 | |  |
|  |  | |  | |  | |  | |  | |  |
|  |  | |  | |  | | **R^2^** | | **R^2^ adjusted** | |  |
|  |  | |  | |  | | 0.49 | | 0.45 | |  |
| **Table S2**. Continued | |  | |  | |  | |  | |  | |
| **LPS act** | **Estimate** | | **SE** | | **P value** | | **lower** | | **upper** | |  |
|  |  | |  | |  | |  | |  | |  |
| Intercept | 188 | | 64 | | 0.005 | | 58 | | 317 | |  |
| Resp. dust | -0.21 | | 0.069 | | 0.004 | | -0.35 | | -0.072 | |  |
| BMI | -337 | | 116 | | 0.006 | | -570 | | -104 | |  |
| Workplace | -0.041 | | 0.11 | | 0.724 | | -0.27 | | 0.19 | |  |
| Stress | 0.073 | | 0.16 | | 0.657 | | -0.26 | | 0.40 | |  |
|  |  | |  | |  | |  | |  | |  |
|  |  | |  | |  | | **R^2^** | | **R^2^ adjusted** | |  |
|  |  | |  | |  | | 0.30 | | 0.24 | |  |
|  |  | |  | |  | |  | |  | |  |
| **CXCL8** | **Estimate** | | **SE** | | **P value** | | **lower** | | **upper** | |  |
| Intercept | -325 | | 116 | | 0.007 | | -557 | | -92 | |  |
| Resp. dust | 0.24 | | 0.13 | | 0.060 | | -0.011 | | 0.49 | |  |
| BMI | 586 | | 209 | | 0.007 | | 166 | | 1006 | |  |
|  |  | |  | |  | |  | |  | |  |
|  |  | |  | |  | | **R^2^** | | **R^2^ adjusted** | |  |
|  |  | |  | |  | | 0.18 | | 0.15 | |  |
|  |  | |  | |  | |  | |  | |  |
| **SAA** | **Estimate** | | **SE** | | **P value** | | **lower** | | **upper** | |  |
| Intercept | -539 | | 111 | | 0.000 | | -762 | | -315 | |  |
| BMI | 998 | | 201 | | 0.000 | | 594 | | 1401 | |  |
| Infection | 0.056 | | 0.20 | | 0.779 | | -0.34 | | 0.45 | |  |
|  |  | |  | |  | |  | |  | |  |
|  |  | |  | |  | | **R^2^** | | **R^2^ adjusted** | |  |
|  |  | |  | |  | | 0.30 | | 0.28 | |  |
|  |  | |  | |  | |  | |  | |  |
| **sST2** | **Estimate** | | **SE** | | **P value** | | **lower** | | **upper** | |  |
| Intercept | 145 | | 61 | | 0.021 | | 23 | | 266 | |  |
| Resp. dust | 0.19 | | 0.086 | | 0.029 | | 0.021 | | 0.36 | |  |
| Quartz | -0.26 | | 0.11 | | 0.020 | | -0.47 | | -0.042 | |  |
| BMI | -252 | | 110 | | 0.026 | | -473 | | -32 | |  |
| Workplace | -0.17 | | 0.11 | | 0.147 | | -0.39 | | 0.060 | |  |
|  |  | |  | |  | |  | |  | |  |
|  |  | |  | |  | | **R^2^** | | **R^2^ adjusted** | |  |
|  |  | |  | |  | | 0.19 | | 0.13 | |  |

*Mixed model analysis performed on the over-shift difference in selected inflammatory biomarkers. The Akaike information criteria was used to select models, by testing the variables respiratory quartz exposure, respiratory dust exposure, BMI, age, smoking status, symptoms of infection last two weeks, mental stress, pre-shift biomarker levels and workplace (foundry A or B). The results are shown for the Box Cox transformed data. N/S: not selected, SE: standard error, lower/upper: lower/upper 95% confidence interval.*

**Table S3**. Mixed model data of selected inflammatory markers for the morning 2^nd^ measurement biomarker levels.

| **IL-1β** | **Estimate** | | **SE** | | **P value** | | **lower** | | **upper** | |  |
| --- | --- | --- | --- | --- | --- | --- | --- | --- | --- | --- | --- |
| Intercept | -1.27 | | 0.58 | | 0.037 | | -2.46 | | -0.085 | |  |
| Quartz | -0.21 | | 0.14 | | 0.152 | | -0.49 | | 0.080 | |  |
| Infection | 0.26 | | 0.21 | | 0.219 | | -0.17 | | 0.69 | |  |
|  |  | |  | |  | |  | |  | |  |
|  |  | |  | |  | | **R^2^** | | **R^2^ adjusted** | |  |
|  |  | |  | |  | | 0.11 | | 0.047 | |  |
|  |  | |  | |  | |  | |  | |  |
| **IL-18** | **Estimate** | | **SE** | | **P value** | | **lower** | | **upper** | |  |
| Intercept | -61 | | 61 | | 0.328 | | -186 | | 65 | |  |
| Quartz | -0.22 | | 0.11 | | 0.049 | | -0.44 | | -0.0013 | |  |
| Age | 0.0044 | | 0.0011 | | 0.000 | | 0.0022 | | 0.0066 | |  |
| BMI | 114 | | 107 | | 0.295 | | -106 | | 335 | |  |
| Stress | 0.044 | | 0.052 | | 0.400 | | -0.062 | | 0.15 | |  |
| Infection | 0.29 | | 0.18 | | 0.120 | | -0.080 | | 0.65 | |  |
|  |  | |  | |  | |  | |  | |  |
|  |  | |  | |  | | **R^2^** | | **R^2^ adjusted** | |  |
|  |  | |  | |  | | 0.4303 | | 0.3163 | |  |
|  |  | |  | |  | |  | |  | |  |
| **IL-1Ra** | **Estimate** | | **SE** | | **P value** | | **lower** | | **upper** | |  |
| Intercept | -169 | | 68 | | 0.019 | | -308 | | -30 | |  |
| BMI | 304 | | 119 | | 0.016 | | 60 | | 549 | |  |
| Workplace | 0.23 | | 0.13 | | 0.097 | | -0.044 | | 0.50 | |  |
|  |  | |  | |  | |  | |  | |  |
|  |  | |  | |  | | **R^2^** | | **R^2^ adjusted** | |  |
|  |  | |  | |  | | 0.2517 | | 0.1982 | |  |
|  |  | |  | |  | |  | |  | |  |
| **ATP** | **Estimate** | | **SE** | | **P value** | | **lower** | | **upper** | |  |
| Intercept | 248 | | 123 | | 0.054 | | -4.44 | | 500 | |  |
| Quartz | -1.08 | | 0.21 | | 0.000 | | -1.52 | | -0.64 | |  |
| Age | 0.0064 | | 0.0021 | | 0.004 | | 0.0022 | | 0.011 | |  |
| BMI | -442 | | 216 | | 0.051 | | -886 | | 2.43 | |  |
|  |  | |  | |  | |  | |  | |  |
|  |  | |  | |  | | **R^2^** | | **R^2^ adjusted** | |  |
|  |  | |  | |  | | 0.52 | | 0.47 | |  |
|  |  | |  | |  | |  | |  | |  |
| **LPS+ATP** | **Estimate** | | **SE** | | **P value** | | **lower** | | **upper** | |  |
| Intercept | 213 | | 112 | | 0.068 | | -17 | | 442 | |  |
| Quartz | -1.26 | | 0.20 | | 0.000 | | -1.68 | | -0.84 | |  |
| Age | 0.0076 | | 0.0019 | | 0.001 | | 0.0037 | | 0.012 | |  |
| BMI | -378 | | 197 | | 0.066 | | -783 | | 27 | |  |
| Workplace | -0.13 | | 0.24 | | 0.592 | | -0.62 | | 0.36 | |  |
| smoking | -0.29 | | 0.14 | | 0.052 | | -0.59 | | 0.0032 | |  |
|  |  | |  | |  | |  | |  | |  |
|  |  | |  | |  | | **R^2^** | | **R^2^ adjusted** | |  |
|  |  | |  | |  | | 0.68 | | 0.61 | |  |
|  |  | |  | |  | |  | |  | |  |
|  | |  | |  | |  | |  | |  | |
|  | |  | |  | |  | |  | |  | |
| **Table S3**. Continued | |  | |  | |  | |  | |  | |
| **LPS act** | **Estimate** | | **SE** | | **P value** | | **lower** | | **upper** | |  |
|  |  | |  | |  | |  | |  | |  |
| Intercept | -0.36 | | 0.93 | | 0.704 | | -2.28 | | 1.56 | |  |
| Resp. dust | -0.37 | | 0.18 | | 0.052 | | -0.74 | | 0.0038 | |  |
| Workplace | -0.95 | | 0.29 | | 0.003 | | -1.54 | | -0.35 | |  |
| Stress | 0.43 | | 0.12 | | 0.001 | | 0.18 | | 0.67 | |  |
| Infection | 0.39 | | 0.35 | | 0.271 | | -0.32 | | 1.11 | |  |
|  |  | |  | |  | |  | |  | |  |
|  |  | |  | |  | | **R^2^** | | **R^2^ adjusted** | |  |
|  |  | |  | |  | | 0.60 | | 0.54 | |  |
|  |  | |  | |  | |  | |  | |  |
| **CXCL8** | **Estimate** | | **SE** | | **P value** | | **lower** | | **upper** | |  |
| Intercept | -275 | | 130 | | 0.044 | | -542 | | -8.02 | |  |
| Resp. dust | 0.37 | | 0.16 | | 0.028 | | 0.042 | | 0.69 | |  |
| BMI | 484 | | 229 | | 0.044 | | 14 | | 954 | |  |
|  |  | |  | |  | |  | |  | |  |
|  |  | |  | |  | | **R^2^** | | **R^2^ adjusted** | |  |
|  |  | |  | |  | | 0.25 | | 0.20 | |  |
|  |  | |  | |  | |  | |  | |  |
| **SAA** | **Estimate** | | **SE** | | **P value** | | **lower** | | **upper** | |  |
| Intercept | -358 | | 163 | | 0.037 | | -693 | | -23 | |  |
| BMI | 657 | | 287 | | 0.030 | | 69 | | 1245 | |  |
| Infection | -0.68 | | 0.43 | | 0.127 | | -1.56 | | 0.21 | |  |
|  |  | |  | |  | |  | |  | |  |
|  |  | |  | |  | | **R^2^** | | **R^2^ adjusted** | |  |
|  |  | |  | |  | | 0.23 | | 0.17 | |  |
|  |  | |  | |  | |  | |  | |  |
| **sST2** | **Estimate** | | **SE** | | **P value** | | **lower** | | **upper** | |  |
| Intercept | 3.59 | | 0.53 | | 0.000 | | 2.51 | | 4.67 | |  |
| Quartz | -0.39 | | 0.13 | | 0.005 | | -0.66 | | -0.13 | |  |
| Workplace | -0.13 | | 0.14 | | 0.363 | | -0.42 | | 0.160 | |  |
|  |  | |  | |  | |  | |  | |  |
|  |  | |  | |  | | **R^2^** | | **R^2^ adjusted** | |  |
|  |  | |  | |  | | 0.32 | | 0.27 | |  |

*Mixed model analysis performed on the morning levels (2^nd^ measurement) of selected inflammatory biomarkers. The Akaike information criteria was used to select models, by testing the variables respiratory quartz exposure, respiratory dust exposure, BMI, age, smoking status, symptoms of infection last two weeks, mental stress and workplace (foundry A or B). The results are shown for the Box Cox transformed data. N/S: not selected, SE: standard error, resp.: respirable, lower/upper: lower/upper 95% confidence interval.*
